# UNet-ECA-Bio: a biologically informed deep learning model for high-throughput micro-phenotyping of rice stem vascular bundles

**DOI:** 10.3389/fpls.2026.1893490

**Published:** 2026-07-28

**Authors:** Jianguo Li, Xiaoying Zhu, Zesheng Wei, Xiaoti Huang, Mingchong Yang, Dandan He, Jiahua Shi, Haoran Li, Huan Wang, Jiada Huang, Jun Shen, Lingqiang Wang

**Affiliations:** 1 Guangxi Sugarcane Bio-breeding Laboratory, State Key Laboratory for Conservation and Utilization of Subtropical Agro-bioresources, College of Agriculture, Guangxi University, Nanning, China; 2 Guangxi Colleges and Universities Key Laboratory of Intelligent Software, Wuzhou University, Wuzhou, China; 3 Queensland Alliance for Agriculture and Food Innovation, The University of Queensland, St Lucia, QLD, Australia; 4 School of Computing and Information Technology, University of Wollongong, Wollongong, NSW, Australia

**Keywords:** deep learning, GWAS, micro-phenotype, rice stem, vascular bundles

## Abstract

Rice stem internal structure is a critical micro-phenotype influencing lodging resistance and yield; however, its analysis remains constrained by labor-intensive manual methods. Here, we present a publicly available dataset of 686 rice stem cross-sections, with 21,027 large vascular bundles (LVBs) and 19,342 small vascular bundles (SVBs) manually annotated. Five deep learning architectures were systematically evaluated, among which UNet-VGG16 achieved the best performance with a mean intersection over union (mIoU) of 87.4% (82.49% for LVBs and 74.41% for SVBs). An improved model, UNet-ECA-Bio, further raised mIoU to 89.32% and SVB IoU to 78.97% by integrating Efficient Channel Attention (ECA) and biologically informed class weighting using an image-level dataset. Leveraging these high-accuracy phenotypic predictions, genome-wide association studies (GWAS) indicated concordance between annotated and predicted traits, with SNP overlap rates of 96% (LVB count: 1,217/1,262), 43% (SVB count: 6/14), 98% (stem area: 122/124), and 100% (cavity area: 3/3) at −log10(p) ≥ 6. Meanwhile, compared with manual annotation (estimated 10–30 minutes per image), the proposed approach processed all 686 images within 10 minutes, representing a >600-fold increase in throughput. We further developed a user-friendly software tool, “Rice_Stem_Pre_V1.1.exe,” for automated phenotyping of 14 stem traits, providing a cost-effective platform for genetic studies of lodging resistance and yield improvement.

## Introduction

Rice (*Oryza sativa* L.) is one of the most important cereal crops, feeding over half of the world’s population (Yuan et al., 2021). Significant efforts were devoted to identifying key genes that may improve rice production by exploring the connection between genotype and phenotype (Fang et al., 2019; Wu et al., 2023; Zhang et al., 2022). Recent advancements in sequencing technologies have significantly improved the ability to genotype a larger population. In contrast, strategies for assessing phenotypic traits have lagged behind advances in genotyping, creating a gap in identifying candidate genes through genome-wide association studies (GWAS) and quantitative trait locus (QTL) mapping. This gap complicates the understanding of trait inheritance, hindering the connection between genetic variation and observable phenotypes (Zhang et al., 2024). Therefore, establishing efficient phenotyping strategies is crucial for enhancing genetic gains (Seck et al., 2023).

Recently, high-throughput platforms have been successfully employed to decipher the genetic basis of developmental and agronomic traits in crops such as rice (Geng et al., 2024), wheat (Gao et al., 2023), and maize (Wang et al., 2022). These platforms generally capture macro-phenotypes such as plant height, leaf size, and panicle growth (Gao et al., 2023; Wang et al., 2022). However, the micro-phenotype, such as the stem’s internal structure, has received less attention due to measurement challenges (Skelton, 2019). Although stem internal structure phenotyping has been achieved in maize stalks using micro-computed tomography (CT), this technology remains inaccessible to many researchers due to its high cost and specialized equipment (Du et al., 2016). In addition, several semi-automated stereo-microscope methods have been reported for measuring the internal structure of stems in sorghum (Fan et al., 2022) and rice (Li et al., 2024). However, these semi-automated methods still rely on manual annotation, making them labor-intensive and time-consuming. A cost-effective, high-throughput method for phenotyping stem internal structures remains unavailable.

Deep learning-based approaches, particularly those leveraging Convolutional Neural Networks (CNNs), are widely used for image-based phenotyping in plant phenomics due to their ability to extract multiple image features (Liu et al., 2019; Miao et al., 2024; Yasrab et al., 2019). In semantic segmentation, UNet (Ronneberger et al., 2015), PSPNet (Zhao et al., 2017), and DeepLab (Chen et al., 2017) have become the mainstream methods and have been used to segment foxtail millet seeds (Miao et al., 2024), rice seedlings (Zhang et al., 2023), maize pests (Kang et al., 2023), and the wheat stripe rust disease index (Deng et al., 2023). Among them, the UNet model has achieved significant success in medical image segmentation (Soulami et al., 2021; Xiao et al., 2020). Given the similarities between medical images and micro-phenotypes—distinct objects within relatively uniform backgrounds—it is highly feasible to transfer segmentation models to stem internal structure analysis. Recently, the You Only Look Once (YOLO) model has been popular for its speed and accuracy and has been widely used in plant phenomics (Jia et al., 2024; Lan et al., 2024; Zhang et al., 2023). YOLOv8, a cutting-edge, state-of-the-art model, achieves high segmentation recognition accuracy and has been used for plant features such as soybean radicle (Wu et al., 2024) and rice disease spots (Yang et al., 2024). With the tremendous success of natural language processing, recent years have witnessed a surge of interest in leveraging transformers for vision tasks (Ali et al., 2023). SegFormer, a simple, efficient, yet powerful framework that unifies Transformers with lightweight multilayer perceptron decoders, has achieved higher accuracy in semantic segmentation and has also been used in crops such as rice and wheat (Qiu et al., 2025; Wang et al., 2024; Xie et al., 2021). Furthermore, a growing number of image-based phenotyping approaches coupled with GWAS have been reported. For instance, an improved U-Net-based pipeline integrated with GWAS has been applied to elucidate the genetic variation underlying temporal salinity responses in wheat (Li et al., 2026). Similarly, a U-Net variant augmented by ASPP and CBAM—to phenotype root anatomical traits across 352 wheat accessions, and subsequent GWAS uncovered 97 QTLs for root tissue architecture, nominating *TaSPL14* as a key regulator (Cheng et al., 2025). In rice, Xu recently demonstrated that high-resolution 3D reconstruction of individual tillers enables extraction of stem-related architectural traits for GWAS, identifying 93 significant SNPs across seven tiller traits (Xu et al., 2025).

To better capture the varying importance of different channels and spatial regions in feature maps, attention mechanisms have been introduced. These mechanisms help models focus on the most informative features, thereby improving classification performance. The Squeeze-and-Excitation Network (SENet) enhances channel-wise feature representation by modeling inter-channel dependencies through global average pooling followed by fully connected layers (Hu et al., 2018). However, the dimensionality reduction used in SENet may weaken the quality of channel attention. To address this issue, the Efficient Channel Attention (ECA) mechanism removes the fully connected layers and directly applies a 1×1 convolution after global average pooling (Wang et al., 2020). This design avoids dimensionality reduction, effectively captures cross-channel interactions, and achieves strong performance with minimal additional parameters. In contrast, the Convolutional Block Attention Module (CBAM) extends attention to both channel and spatial dimensions, enabling the model to refine feature maps more comprehensively (Woo et al., 2018).

In this study, we seek to develop a cost-effective approach for rapidly phenotyping the internal structure of rice stems. First, we made an open-source dataset of stem internal structures. We compared the performance of five deep learning models with various backbones for semantically segmenting the internal structure of the rice stem. Second, we enhance the model’s ability to accurately delineate micro-phenotypes, particularly vascular bundles, and validate its application in genome-wide association studies. Finally, we present a user-friendly application of the optimized model.

## Materials and methods

### Experimental and technical design

To investigate the internal structure of rice stems, this study applied deep learning-based semantic segmentation. Cross-sectional samples of the main stem were collected at the rice heading stage, when 50% of the individual plants had produced spikes in a line. The middle sections of the second inverted internodes were then sliced, stained, and imaged using an OLYMPUS CORPORATION SZ2-ILST microscope. These images were annotated and converted into masks. The original slices and their corresponding masks were used as the dataset to train five deep-learning models: PSPNet, DeepLabV3plus, UNet, SegFormer, and YOLOv8. Finally, the most accurate model was chosen for the semantic segmentation of the rice stem’s internal structure.

### Diversity of plant materials

A total of 686 rice stem slices were collected based on 165, 120, and 17 rice accessions in 2020, 2021, and 2024, respectively (**Supplementary Table 1**). In detail, there were 488 stem images for 165 accessions in 2020, 121 stem images for 120 accessions in 2021, and 77 stem images for 17 accessions in 2024. The association panel used for GWAS comprised 165 rice accessions planted in 2020 in Nanning, China, with three biological replicates per accession. The field management followed local agricultural practices.

### Microscopy of stem internal structure

The cross-section samples of the main stem were collected at the heading stage. Firstly, the middle sections of the second inverted internodes from rice plants were manually cut into slices 0.2-0.4 mm thick. Subsequently, the stem slices were stained for 15 seconds using a solution of 5% phloroglucinol [ethyl alcohol: water = 95:5, (v/v)] and concentrated hydrochloric acid. The slices were then photographed with an OLYMPUS CORPORATION SZ2-ILST microscope, with a resolution of 3584 px × 2748 px.

### Manually annotating images of rice stem slices

The software LabelMe (v5.2.1) was used to annotate the vascular bundles, stem, and cavity in images, and the annotations were subsequently saved in JSON format. The labels comprised four categories: SVB, LVB, stem, and cavity. For convenience, the tags for SVB, LVB, stem, and cavity were renamed “small”, “big”, “out”, and “in” within the LabelMe software. Afterward, the images were converted into masks using the LabelMe script (Russell et al., 2008). Further, the random.seed(0) was used to generate datasets among different models. The dataset was randomly split into a training set and a validation set in a 9:1 ratio, with 617 images for training set and 69 images for validation set. This split was performed at the image level; consequently, slices from the same accession may appear in both the training and validation sets.

### UNet, DeepLabV3plus, and PSPNet, SegFormer and YOLOv8 parameters

Firstly, each image is re-scaled into $x\;\in \;{\mathbb{R}}^{512\times 512\times 3}$ as the model’s input. During training, the Adam optimizer is used with a cosine learning-rate decay strategy for UNet, while AdamW is used for SegFormer. For DeepLabV3plus and PSPNet, the optimizer is changed to stochastic gradient descent (SGD). For YOLOv8, the optimizer was automatically set. All five models are trained for 200 epochs. Mean Intersection over Union (mIoU) was chosen to evaluate the accuracy of the overall target regions:


$$mIoU=\;\frac{1}{K}\sum_{i=1}^{K}\frac{TP}{TP+FN+FP}$$

Notes: TP: true positive, FN: false negative, FP: false positive.

Ultimately, R-squared (R²), Root Mean Square Error (RMSE), and Mean Absolute Error (MAE) were used in R version 4.0 to assess model performance on the dataset, and the R package ggplot2 (3.4.2) was used to visualize the results. We train all models on a single NVIDIA RTX 4060 Ti (8 GB) GPU. The experiments are implemented in Python 3.8, with PyTorch (2.3.1 + cu118). PyInstaller (6.11.1) was employed to package the script into Rice_Stem_Pre_V1.1.exe. The Integrated Development Environment used was Visual Studio Code (1.92.2).

### Genome-wide association study

The genotype data of the rice association panel were downloaded from https://snp-seek.irri.org (Alexandrov et al., 2015). PLINK (1.9) was used to extract genotype information and remove markers with minor allele frequency (MAF) < 0.01 and per-marker missingness> 0.01 (Purcell et al., 2007). As a result, a total of 2.3M SNPs were obtained. Subsequently, a GWAS was conducted using the fixed and random model circulating probability unification (FarmCPU) model with rMVP (https://github.com/xiaolei-lab/rMVP) (Yin et al., 2021). The R package CMplot (version 4.3.1) was used to generate the Manhattan plot from the GWAS results (Yin et al., 2021). The significance threshold was determined using GEC software (Genetic type I Error Calculator)(https://pmglab.top/gec/#/), which estimated the effective number of independent tests as 349,941.57 from 2,298,054 observed SNPs. The corresponding genome-wide suggestive significance level was 1/Meff = 2.86 ×^-6^ (−log10(p) ≈ 5.54), rounded to −log10(p) = 6 for convenient visualization in Manhattan plots. The FarmCPU model included a kinship matrix and the first two principal components to control for population structure. The annovar software were used to annotate the significant SNPs and https://venyao.xyz/funRiceGenes/was used to obtain the gene information (Wang et al., 2010). LDBlockshow(1.41) was used to visualize the linkage disequilibrium of the significant SNPs (Dong et al., 2021).TBTools(v2.154) was used to visualize the distribution of QTLs (Chen et al., 2020).

## Results

### Performance benchmarking of five deep learning architectures

**Figure 1:**
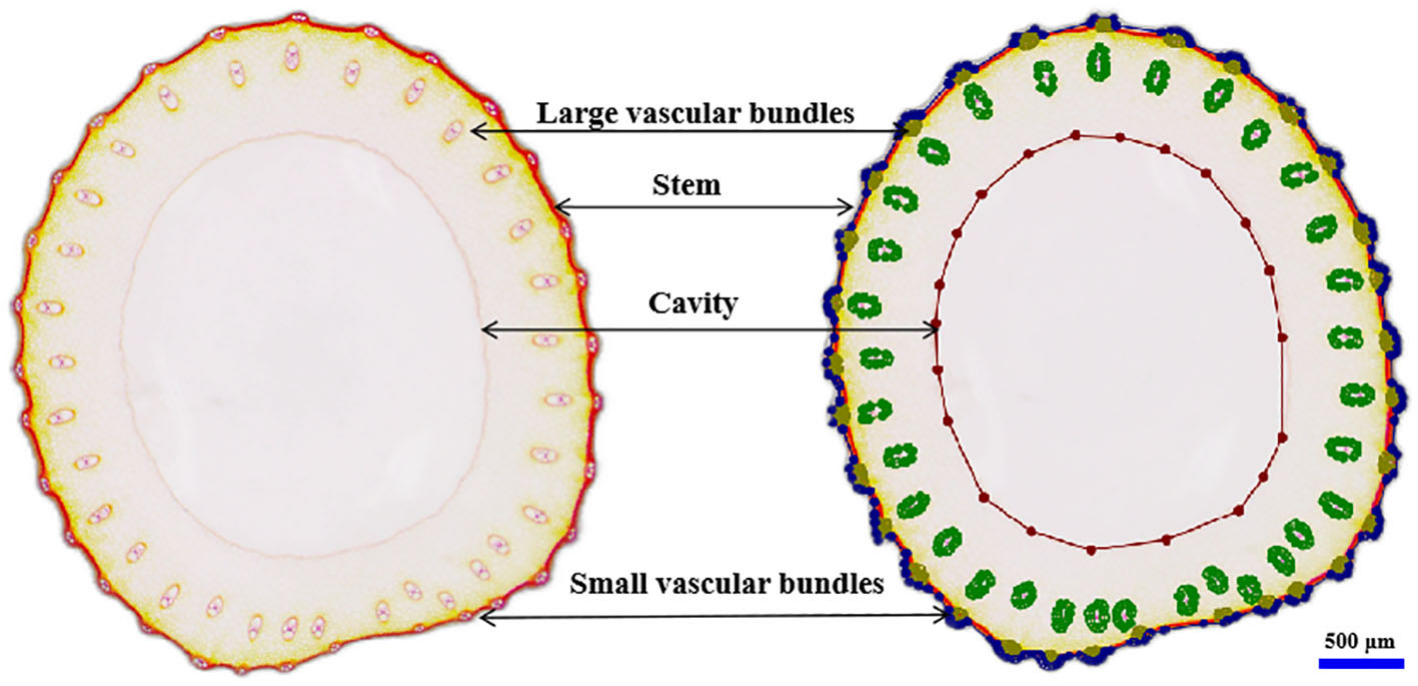
The comparison of rice stem slices before and after annotation.

A total of 686 rice stem slices were collected from 165, 120, and 17 rice accessions in 2020, 2021, and 2024, respectively. These stem slices were treated with phloroglucinol and concentrated hydrochloric acid, and then magnified under the microscope. Then, the diverse stem slices dataset was annotated and converted into mask images using LabelMe software (**Figure 1**). The dataset is available for download as open source. In detail, a total of 21027, 19342, 686, and 686 LVB, SVB, stems, and cavities were labeled.

**Figure 2:**
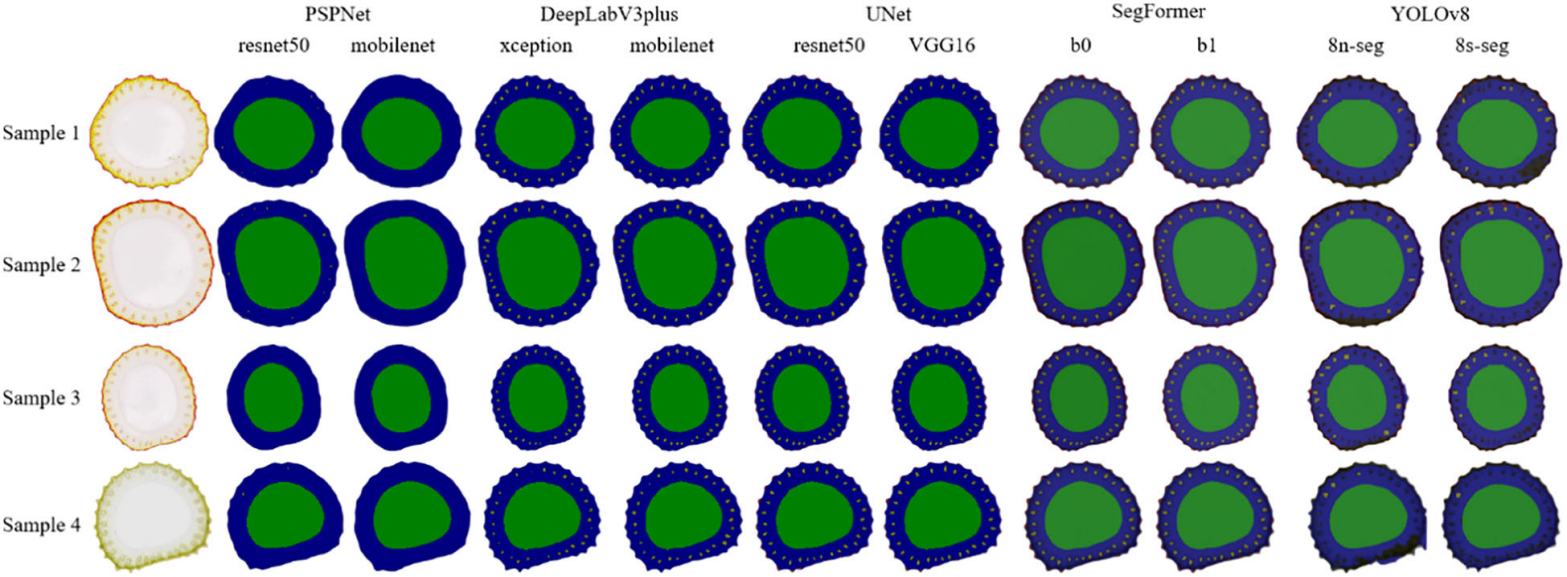
Comparison of five models with different backbones. The samples 1, 2, and 3 were stem slices stained for 15 seconds using a solution of 5% phloroglucinol [ethyl alcohol: water = 95:5, (v/v)] and concentrated hydrochloric acid. Sample 4 consisted of stem slices without pretreatment.

Subsequently, the untreated slices and their corresponding masks were used to train five deep-learning models with various backbones (UNet-resnet50, UNet-VGG16, DeepLabV3plus-mobilenet, DeepLabV3plus-xception, PSPNet-Mobilenet, PSPNet-resnet50, SegFormer-b0, SegFormer-b1, YOLOv8-8n-seg, and YOLOv8-8s-seg) for segmenting the internal structure of the rice stem (**Figure 2**). As a result, PSPNet showed the lowest performance, with mean Intersection over Union (mIoU) values of 45.67% for PSPNet-mobilenet and 48.01% for PSPNet-resnet (**Table 1**). Specifically, PSPNet-mobilenet achieved Intersection over Union (IoU) values of 96.72% for cavity, and 85.94% for stem, while PSPNet-resnet showed IoUs of 97.42%, and 87.75%, respectively. However, PSPNet struggled with segmenting vascular bundles, yielding IoUs of 0.49% for SVB and 6.37% for LVB in the PSPNet-resnet50 model. The YOLOv8 model showed performance similar to PSPNet, achieving mIoUs of 45.91% and 38.09% for YOLOv8-8n-seg and YOLOv8-8s-seg, respectively. In contrast, DeepLabV3plus-mobilenet and DeepLabV3plus-xception outperformed PSPNet and YOLOv8, with mIoU of 75.87% and 72.42%, respectively (**Figure 2**). DeepLabV3plus models notably excelled in segmenting SVB and LVB, with IoU values of 42.51% and 70.97% in DeepLabV3plus-mobilenet, and 34.12% and 66.69% in DeepLabV3plus-xception. The SegFormer outperformed DeepLabV3plus, achieving mIoUs of 82.73% and 84.21% for SegFormer-b0 and SegFormer-b1, respectively. Both SegFormer-b0 and SegFormer-b1 outperformed DeepLabV3plus in SVB and LVB segmentation. Among all the models tested, UNet showed the best performance, achieving mIoUs of 86.54% for UNet-resnet50 and 87.40% for UNet-VGG16. The UNet-VGG16 model showed improvement in segmenting SVB and LVB, with IoU values of 74.41% and 82.49%, respectively. As a result, the UNet model with the VGG16 backbone was selected for further study.

**Table 1:** Comparison of five models with different backbones in semantic segmentation of stem internal structure.

Features	PSPNet (mobilenet)	PSPNet (resnet50)	DeepLabV3plus (mobilenet)	DeepLabV3plus (xception)	UNet (resnet50)	UNet (VGG16)	SegFormer (b0)	SegFormer (b1)	YOLOv8 (8n-seg)	YOLOv8 (8s-seg)
SVB	0	0.49	42.51	34.12	72.33	74.41	63.54	66.82	5.7	3.73
Cavity	96.72	97.42	97.52	97.39	97.95	97.93	97.77	97.82	86.2	75.84
LVB	0	6.37	70.97	66.69	81.32	82.49	76.25	78.4	20.15	12.27
Stem	85.94	87.75	92.49	91.47	94.55	94.77	93.34	93.86	71.57	60.5
mIoU(%)	45.67	48.01	75.87	72.42	86.54	87.40	82.73	84.23	45.91	38.09

### Optimizing UNet-VGG16 for enhanced segmentation precision in rice stem internal structure

To optimize the deep learning model for rice stem segmentation, various parameters in the UNet-VGG16 model were systematically adjusted. First, different initial learning rates (0.1, 0.01, 0.001, 0.0001) were tested, yielding mIoUs of 87.45%, 87.46%, 87.42%, and 87.40%, respectively, indicating minimal impact on segmentation performance (**Supplementary Table 2**). Next, different batch sizes (1, 2, and 4) were explored, yielding mIoUs of 87.48%, 87.40%, and 87.33%, respectively, suggesting that batch size had a negligible effect (**Supplementary Table 3**). Based on these findings and available computational resources, a batch size of 1 and an initial learning rate of 0.0001 were selected for subsequent experiments.

**Figure 3:**
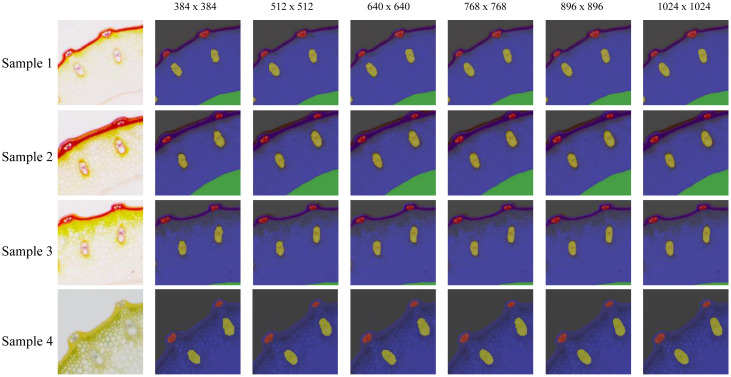
Comparison of semantic segmentation among different input sizes in the UNet-VGG16 model. The red masks represent SVB. The yellow masks represent LVB. The green masks represent the cavity area. The samples 1, 2, and 3 were stem slices stained for 15 seconds using a solution of 5% phloroglucinol [ethyl alcohol: water = 95:5, (v/v)] and concentrated hydrochloric acid. Sample 4 consisted of the stem slice without pretreatment.

Among the stem internal traits, we observed that the IoUs for SVB and LVB were lower than those for the stem and cavity, likely due to the smaller size of the vascular bundles. To enhance performance in vascular bundle segmentation, we tested various image input sizes (384×384, 512×512, 640×640, 768×768, 896×896, and 1024×1024), yielding mIoUs of 86.28%, 87.48%, 88.24%, 88.65%, 88.80%, and 88.97%, respectively (**Figure 3**; **Supplementary Table 4**). Besides, we compared the mIoU and IoU for SVB across varying input sizes (**Supplementary Figure 1**). As a result, we found that mIoU consistently achieves higher scores, stabilizing around 90% at an input size of 1024×1024 pixels, and IoU for SVB also shows a steady improvement, reaching approximately 80% at the same input size. Additionally, different training epochs (50, 100, 150, and 200) were evaluated, with mIoUs of 84.74%, 86.78%, 88.32%, and 88.97%, suggesting that higher epochs improved performance (**Supplementary Table 5**). Notably, while the IoUs for background, cavity, and stem showed minimal differences, the IoU for vascular bundles—particularly SVB—showed substantial improvement. Based on the resource consumption and segmentation precision, UNet-VGG16 with a 1024×1024 input size was selected for further analysis.

### Biologically-informed attention UNet enhances the semantic segmentation of rice stem vascular bundles

To further enhance segmentation performance for SVB in rice stem slices, we incorporated attention modules into the UNet architecture’s downsampling path. We compared three attention-based variants, UNet-CBAM, UNet-SEBlock, and UNet-ECANet, against the baseline UNet. The mIoU for UNet, UNet-CBAM, UNet-SEBlock, and UNet-ECANet were 88.97%, 89.06%, 89.11%, and 89.15%, respectively. The corresponding IoU for SVB were 78.21%, 78.32%, 78.50%, and 78.61%. Additionally, improvements were observed in the IoU of LVB, with values of 84.44%, 84.57%, 84.56%, and 84.69%, respectively. Initially, equal class weights (1:1:1:1 for SVB: LVB: stem: cavity) were used during training. To specifically improve SVB segmentation, we increased the weights of SVB to 2 and 3, resulting in class weight ratios of 2:1:1:1 and 3:1:1:1, respectively. This adjustment led to progressive gains in IoU for SVB: 78.61%, 78.78%, and 78.81% for weights 1, 2, and 3, respectively. However, while the mIoU for UNet-ECA2 reached 89.23%, further increasing the weights to 3 (UNet-ECA3) did not improve overall performance, as it led to a decline in the IoU of the LVB, cavity, and stem regions. To address this trade-off, we introduced UNet-ECA-Bio (**Supplementary Figure 2**), which employs biologically informed class weights based on the frequency and area of each structure (SVB: LVB: cavity: stem = 2.29:1.36:0.22:0.13). This configuration achieved the highest overall mIoU of 89.32%, with consistent improvements across all categories, particularly in SVB segmentation (78.97%) (**Table 2**).

**Table 2:** Segmentation performance of attention-based UNet models with different SVB weights.

Features	UNet	UNet-SE	UNet-CBAM	UNet-ECA1	UNet-ECA2	UNet-ECA3	UNet-ECA-Bio
SVB	78.21	78.32	78.5	78.61	78.78	78.81	78.97
LVB	84.44	84.57	84.56	84.69	84.84	84.83	84.94
Cavity	97.98	98.08	98.07	98.04	98.03	98.02	98.11
Stem	95.25	95.26	95.31	95.25	95.25	95.21	95.25
mIoU(%)	88.97	89.06	89.11	89.15	89.23	89.22	89.32

UNet-SE represents UNet-SEBlock. ECA1, ECA2, and ECA3 represent the weights of SVB 1, 2, and 3.

### Model performance in the segmentation of rice stem internal structures

**Figure 4:**
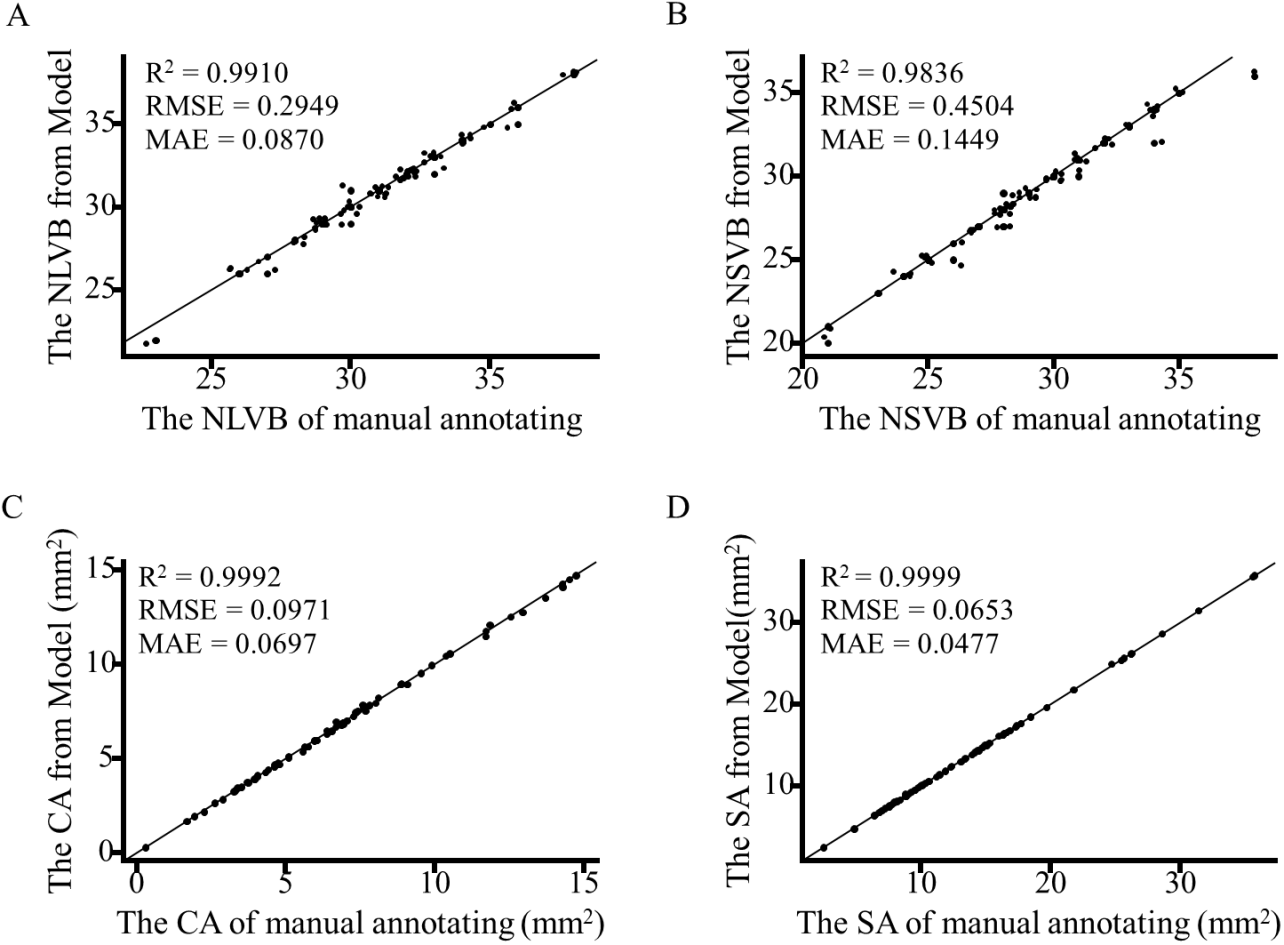
Prediction accuracy of stem structural traits using the UNet-ECA-Bio model (n=69). **(A)** Number of LVB. **(B)** Number of SVB. **(C)** Cavity area (mm^2^). **(D)** Stem area (mm^2^). R^2^, RMSE, and MAE represent the coefficient of determination, root mean square error, and mean absolute error, respectively.

To evaluate the model’s performance, the UNet-ECA-Bio was applied to the validation set, focusing on four specific traits: number of SVB, number of LVB, stem area, and cavity area. The predicted values were compared with the annotated counterparts. The results showed an excellent fit, with a coefficient of determination (R²) of 0.9910, root mean squared error (RMSE) of 0.2949, and mean absolute error (MAE) of 0.0870 for the number of LVB (**Figure 4A**). For the number of SVB, the R² was 0.9836, with an RMSE of 0.4504 and an MAE of 0.1449 (**Figure 4B**). The cavity area achieved an R² of 0.9992, an RMSE of 0.0971, and an MAE of 0.0697 (**Figure 4C**). Likewise, the stem area showed near-perfect accuracy, with an R² of 0.9999, an RMSE of 0.0653, and an MAE of 0.0477 (**Figure 4D**). These results indicate strong agreement between predicted and annotated values on the current validation set using an image-level split. The high R² values for stem and cavity areas are consistent with their IoU exceeding 97%, as large homogeneous regions with clear boundaries are inherently easier to segment accurately.

Beyond rice, the UNet-ECA-Bio model’s generalizability was tested on 35 wheat stem slices, which possess a similar vascular bundle architecture. Despite being trained exclusively on rice data, the model achieved a promising mIoU of 73.59% on wheat. Specifically, the IoUs for LVB and SVB reached 63.38% and 49.02%, respectively (**Supplementary Figure 3**). While these metrics are lower than those achieved in rice, visual inspection supports that the model effectively captures the core anatomical features of wheat stems. This cross-species success directly addresses concerns about model transferability and highlights its potential as a common tool for the micro-phenotype of cereal crop stems. It should be also noted that this cross-species evaluation was limited to 35 wheat slices with visual inspection only. A comprehensive evaluation across diverse cereal crops is needed to establish broader applicability.

### High-throughput analysis of rice stem internal structure: An accessible and cost-free approach

**Figure 5:**
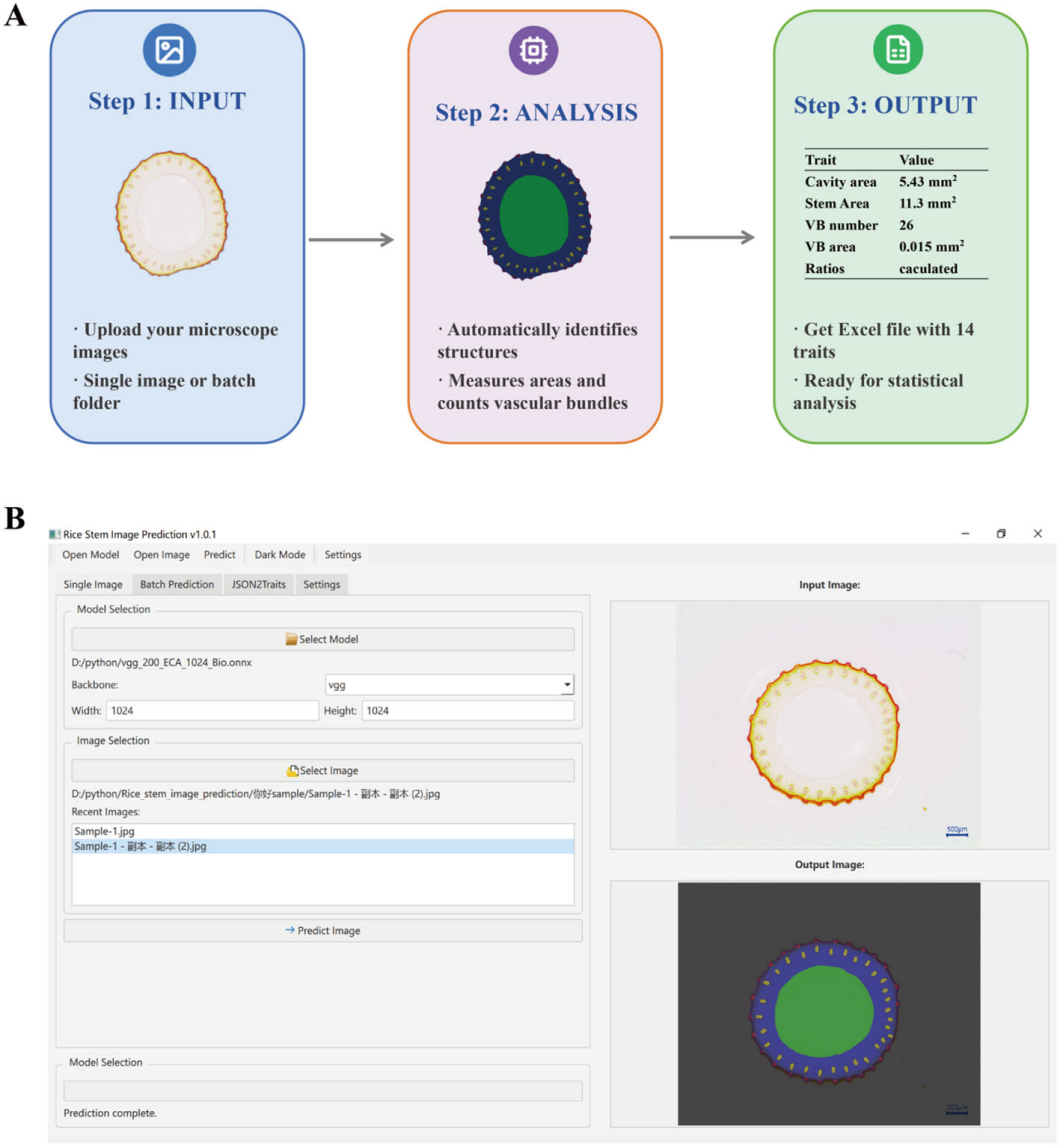
Overview of the rice stem image prediction workflow. **(A)** Workflow pipeline for predicting stem traits from microscopic images. **(B)** Graphical user interface (GUI) for prediction, visualization, and JSON-based result export.

To enhance accessibility and facilitate widespread adoption, we converted the UNet-ECA-Bio model into ONNX format, which is publicly available on Google Drive. We developed two user-friendly implementations: (1) a web-based interface hosted on Hugging Face (https://huggingface.co/spaces/lee214/Rice_stem_prediction), and (2) a standalone executable application (Rice_Stem_Pre_V1.1.exe) for offline analysis. The Rice_Stem_Pre_V1.1.exe application provides an intuitive workflow for automated extraction of stem traits. The analysis workflow follows three sequential steps (**Figure 5A**). Step 1 (INPUT): users upload microscope images of rice stem cross-sections, either individually or as batch folders. Step 2 (ANALYSIS): The model automatically identifies and segments different structures. Step 3 (OUTPUT): The software generates quantitative measurements for 14 stem structural traits, including cavity area, stem area, vascular bundle number, vascular bundle area, and calculated ratios. The software interface of Rice_Stem_Pre_V1.1.exe is shown in **Figure 5B**. The left panel contains controls for model selection (backbone: vgg, dimensions: 1024×1024), image input, and batch processing options. The right panel displays both the input image (top), showing the original rice stem cross-section, and the output image (bottom), showing the color-coded segmentation results. Users simply select the directory containing stem images and specify the scale bar length in millimeters to calibrate measurements.

To support quality control and manual refinement, the software also exports predictions as JSON files, which can be converted to trait measurements using the integrated JSON2Traits function. This automated approach dramatically reduces analysis time compared to conventional manual annotation methods. Traditional protocols using Photoshop, LabelMe, or ImageJ typically require 10–30 minutes per image. In contrast, our UNet-ECA-Bio model processed 686 stem images in approximately 10 minutes—representing a >600-fold increase in throughput. This efficient, cost-effective solution removes a major bottleneck in large-scale phenotyping of the internal structure of rice stems.

### Assessing the concordance between annotated and predicted stem traits in genome-wide association studies

**Figure 6:**
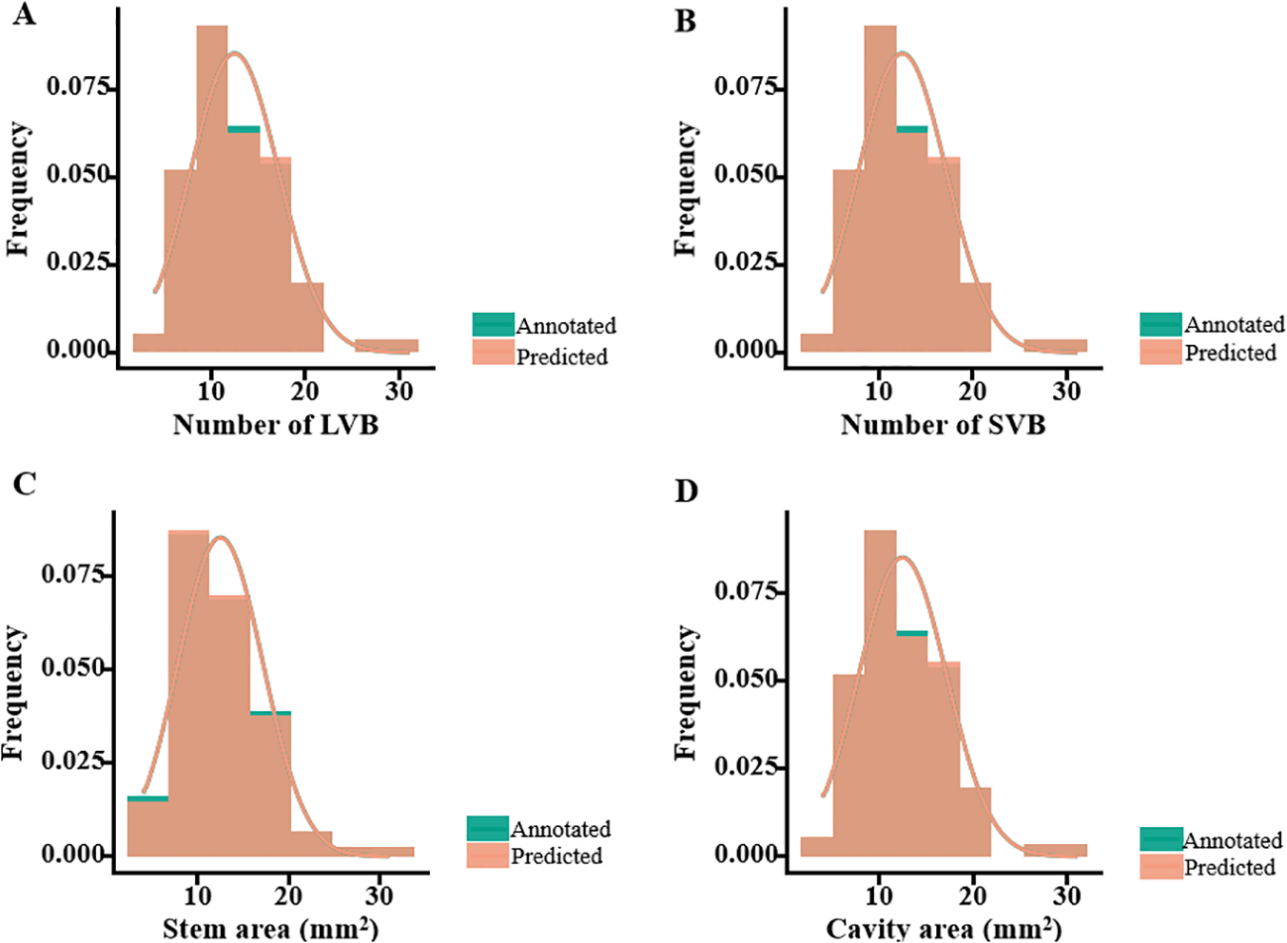
Distribution of annotated and predicted stem structural traits. **(A)** The number of LVB from the UNet-ECA-Bio model and the number of LVB from manual annotation. **(B)** The number of SVB from the UNet-ECA-Bio model and the number of SVB from manual annotation. **(C)** The stem area from the UNet-ECA-Bio model (mm^2^) and the stem area of manual annotation (mm^2^). **(D)** The cavity area from the UNet-ECA-Bio model (mm^2^) and the cavity area of manual annotation (mm^2^).

To evaluate whether the model-predicted traits preserve genetic signals comparable to those from manual annotation, we compared GWAS results between annotated and predicted values. In detail, we compared annotated and predicted values using GWAS based on 165 rice accessions collected in 2020. Both the annotated and predicted stem structure-related traits exhibited similar, approximately normal distributions (**Figure 6**). Specifically, the mean number of LVB was 30.91 for the annotated values and 30.89 for the predicted values, while the mean for SVB was 28.43 and 28.45, respectively (**Supplementary Table 6**). The mean values were 6.14 mm² (predicted) and 6.14 mm² (annotated) for the cavity area, and 12.49 mm² and 12.50 mm² for the stem area, respectively, for the predicted and annotated data. Furthermore, the coefficients of variation for predicted and annotated traits were comparable, indicating that the phenotypic variation captured by the model is consistent with that of the manually annotated data. These results demonstrate a high consistency between the annotated and predicted stem structural traits.

**Figure 7:**
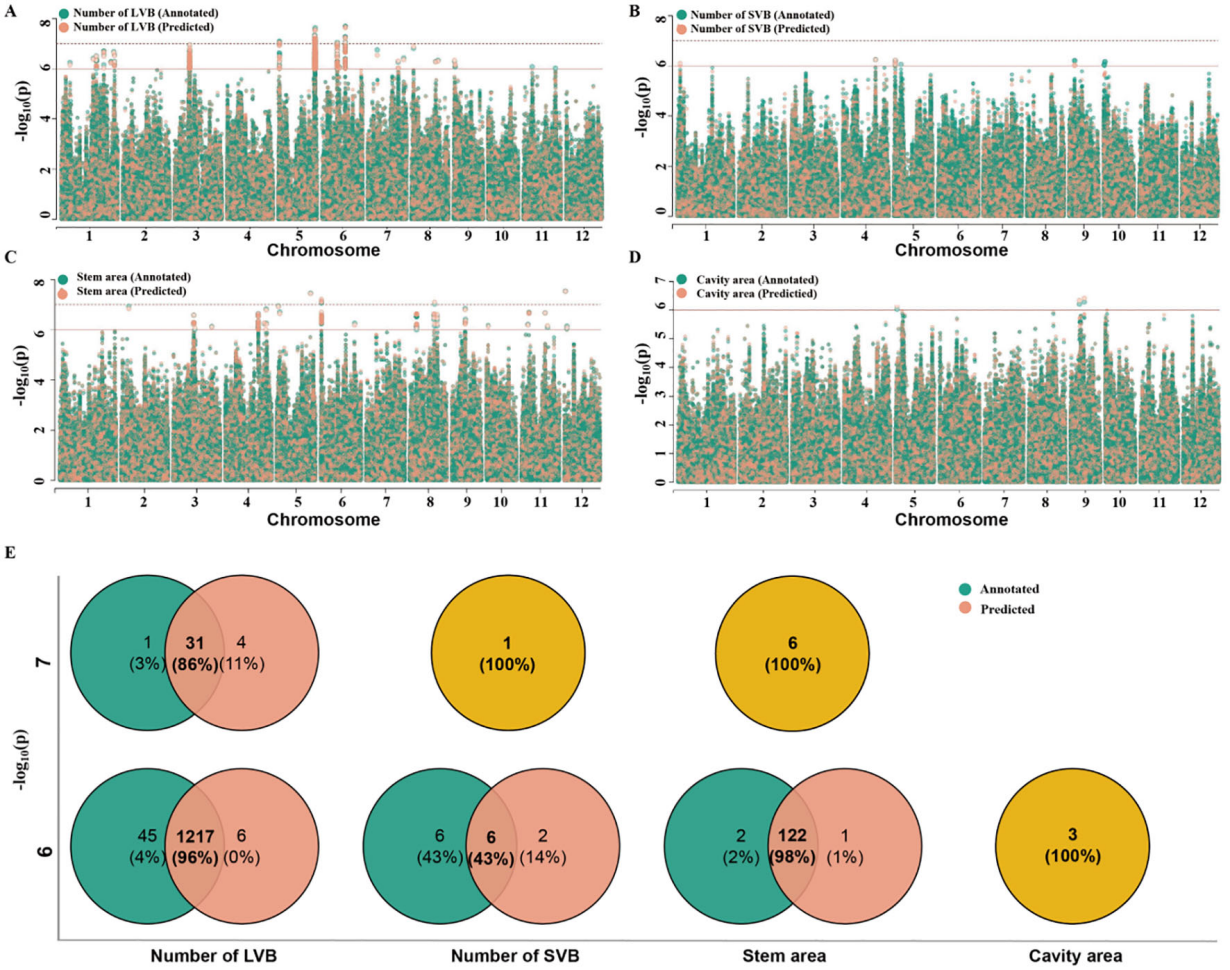
Comparison of GWAS results between annotated and predicted rice stem internal structural traits. **(A)** Manhattan plot of annotated and predicted number of LVB. **(B)** Manhattan plot of annotated and predicted number of SVB. **(C)** Manhattan plot of annotated and predicted stem area (mm^2^). **(D)** Manhattan plot of annotated and predicted cavity area (mm^2^). **(E)** The significant SNPs comparison of annotated and predicted stem internal structure traits with different significant thresholds. The yellow circle represents all SNPs shared between annotated and predicted traits.

Next, we used the FarmCPU model to perform genome-wide association studies (GWAS) for four traits: the number of SVBs, the number of LVBs, the stem area, and the cavity area. The QQ plots for all traits are shown in **Supplementary Figure 4**. The significance threshold was set at a -log_10_(p-value) of 6 based on the GEC software suggested p-value. As a result, 1262 and 1223 significant SNPs were identified for the annotated and predicted number of LVB, respectively, with 1217(96%) significant SNPs being co-detected (**Figure 7A**). For SVB, 12 and 8 significant SNPs were found, with 6(43%) co-detected (**Figure 7B**). Regarding stem area, 124 and 123 significant SNPs were identified for the annotated and predicted values, with 122(98%) co-detected (**Figure 7C**). Finally, three significant SNPs were identified for both the annotated and predicted cavity areas, all of which were co-detected (**Figure 7D**).

Furthermore, we compare GWAS results for annotated and predicted traits across different significance thresholds. When the significance threshold was set at -log10(p-value) = 7, we identified 32 and 35 significant SNPs for the annotated and predicted counts of LVB, respectively, with 31(86%) SNPs co-detected (**Figure 7E**). For the number of SVB and stem area, all significant SNPs were co-detected in the annotated and predicted values. These findings demonstrate that the UNet-ECA-Bio model achieves high accuracy in GWAS applications. In addition, maintaining a conserved significance threshold enhances the accuracy of results derived from high-throughput phenotyping of rice stem internal structures in genome-wide association studies.

**Figure 8:**
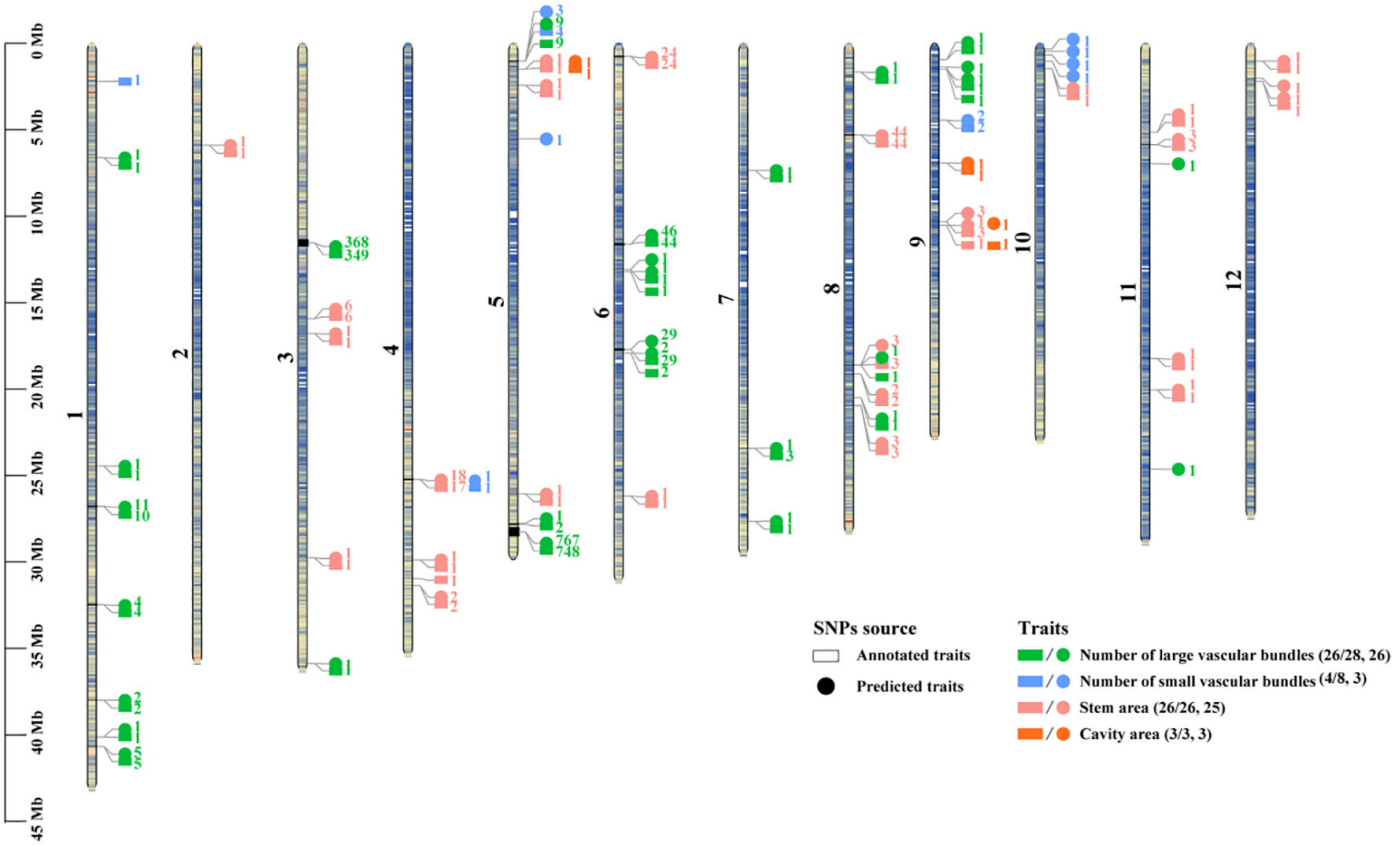
Genome-wide distribution of QTLs associated with vascular bundles, stem area, and cavity area. Colored markers indicate SNPs linked to different traits: green for the number of LVB, blue for the number of SVB, pink for the stem area, and orange for the cavity area.

We further compared the results of annotated and predicted traits at the QTL level by merging SNPs within a 100kb window. The results revealed a high degree of consistency between annotated and predicted traits across all stem internal structural phenotypes. Specifically, 26 and 28 QTLs were identified for the annotated and predicted number of LVB, respectively, with 26 QTLs co-detected. For SVB, 4 and 8 QTLs were detected, with 3 shared QTLs. For the stem area, both annotated and predicted traits identified 26 QTLs, 25 of which were co-detected. Furthermore, for the cavity area, 3 QTLs were identified in both annotated and predicted traits, all of which overlapped (**Figure 8**). These findings demonstrate strong concordance between annotated and model-predicted traits at both the SNP and QTL levels, thereby validating the accuracy and applicability of the proposed deep learning model.

### Functional annotation and pathway analysis of significant SNPs and candidate Genes

To gain a deeper understanding of the significance of SNPs, we performed structural and functional annotations. The significant SNPs could be grouped into nine categories. Among them, 1267(46%) significant SNPs were located in intergenic regions, while 713(26%) significant SNPs were found in the upstream and downstream regions of genes. Additionally, 361(13%) significant SNPs were located within introns, and 295(11%) were in exons (**Supplementary Table 7**). Based on the significant SNPs, 269 genes were annotated, with 250 genes annotated more than twice. Notably, 85 genes were annotated more than ten times. Among these, two auxin response factor (ARF) genes were identified. In particular, LOC_Os05g48870 (*OsARF15*), which encodes auxin response factor 15, was the most frequently annotated gene (285 times), and a targeted mutation of *OsARF15* alters metaxylem vessel size (Reeger et al., 2021). Besides, *WOX12* (LOC_Os05g48990), a member of the WUSCHEL-related homeobox were annotated and WOX genes are associated with stem cell maintenance, cell division and differentiation. In addition, LOC_Os05g48850(*OsSND2*), annotated 71 times, plays a role in cellulose synthesis and biomass production (Ye et al., 2018). LOC_Os05g48880(*OsMMS21*), annotated 56 times, regulates the proliferation of stem cells in rice (Jiang et al., 2021).

To further investigate the potential molecular mechanisms underlying stem structural traits, the 269 annotated genes were analyzed using the STRING database. A total of 57 genes were mapped and functionally annotated. Gene Ontology (GO) enrichment analysis of cellular components revealed significant enrichment in intracellular membrane-bounded organelle (GO:0043231), intracellular anatomical structure (GO:0005622), and cellular anatomical entity (GO:0110165). Using K-means method, 26 genes of the 57 genes were grouped into three clusters (**Supplementary Figure 5**). Cluster 1, comprising 17 genes, was predominantly associated with WOX12-related pathways. Cluster 2 included 7 genes while cluster 3 contained 2 genes that were linked to galactose metabolism. These findings suggest that both the WOX12-related regulatory network and the auxin signaling pathway play crucial roles in the development of the internal structure of the rice stem.

## Discussion

### Design rationale and comparison with existing methods

UNet-ECA-Bio incorporates two innovations — ECA and biologically informed class weighting. ECA adaptively recalibrates channel-wise feature responses through local cross-channel interaction without dimensionality reduction, which distinguishes it from approaches such as SENet (Hu et al., 2018). In deeper network layers, coarse semantic patterns tend to dominate while fine-grained details are suppressed — a known limitation for small-object segmentation. SVBs, which occupy few pixels and are easily overshadowed by larger structures, are particularly vulnerable to this effect. ECA mitigates the issue by preserving informative channels that carry boundary and texture cues relevant to small vascular bundles. The biologically informed class weighting addresses the extreme pixel-level class imbalance inherent in stem cross-section images, where stem and cavity constitute the majority of pixels while SVBs and LVBs occupy a small fraction. Without reweighting, gradient contributions from minority classes are dominated by majority classes. Our weighting scheme assigns higher penalties to SVB and LVB misclassifications based on their relative frequency and area proportion, ensuring the model allocates sufficient representational capacity to vascular bundle features. Because the weights reflect actual anatomical proportions, the approach is interpretable and transferable to other species.

Current methods for phenotyping rice stem internal structures range from fully manual to semi-automated approaches. Manual annotation using LabelMe requires 10–30 min per image. Semi-automated pipelines such as LabelmeP rice reduce some of this burden but still require substantial manual segmentation (Li et al., 2024). More recently, Zhu developed Rice-SVBDete, a YOLOv8-based detector that achieves a detection IoU of 0.651 and mAP@.5 of 0.732 across 1,091 images (Zhu et al., 2025). While effective at localizing individual bundles, its bounding-box output cannot quantify bundle shape, area, or boundary morphology. In contrast, UNet-ECA-Bio provides pixel-level semantic segmentation (mIoU = 89.32%, SVB IoU = 78.97%), enabling direct measurement of bundle area, perimeter, and density — traits essential for GWAS. The two approaches are complementary: detection prioritizes speed and localization, while segmentation provides richer morphological detail. Compared with micro-CT imaging (Du et al., 2016) which requires expensive equipment, our method needs only a standard stereo-microscope, making it accessible to most breeding laboratories. The trade-off is that CT provides three-dimensional structural information, whereas our 2D approach targets traits — vascular bundle count, area, and density — that are well established as biologically informative in GWAS. To our knowledge, UNet-ECA-Bio is the first fully automated pipeline from rice stem cross-section images to GWAS-ready phenotypes.

### Genetic concordance, limitations, and future work

The GWAS comparison between annotated and predicted traits serves a specific methodological purpose: to verify that automated phenotyping does not systematically distort genetic signals. The SNP overlap rates indicate that for these traits, model predictions preserve the genetic associations found through manual annotation. UNet-ECA-Bio improved SVB IoU from 78.21% to 78.97%, yet the GWAS concordance was the lowest among the four traits (43%; 6/14 SNPs). This suggests that current SVB segmentation accuracy, while improved, remains insufficient for reliable GWAS-level genetic dissection. We therefore recommend cautious interpretation of predicted SVB counts in genetic studies and identify further improvement of SVB segmentation — through higher-resolution imaging, refined attention mechanisms, or dedicated training strategies — as a key direction for future work.

Several methodological considerations should be noted. First, the train/validation split was performed at the image level; because each accession may contribute multiple biological replicates, slices from the same accession can appear in both sets. While each slice represents a unique physical cross-section, this design may lead to optimistic generalizability estimates, particularly for traits with low within-accession variability. Accession-level or year-wise cross-validation would strengthen future studies. Second, the annotated-versus-predicted GWAS comparison was conducted on the same 165-accession panel from which both phenotypes were derived. This tests concordance between measurement methods rather than generalization to an independent population — a standard design for method-validation studies but one that would benefit from independent replication. Third, the wheat cross-species evaluation was limited to 35 slices with visual inspection only; broader evaluation across diverse cereal crops is required before general applicability can be established. Fourth, the genomic inflation factor in our GWAS ranged from 1.31 to 1.60. Although we employed the FarmCPU model with kinship and PCA covariates to mitigate confounding effects, we acknowledge that residual inflation remains a methodological caveat that should be considered when interpreting these results. Fifth, quantifying the extent to which these gains exceed run-to-run variation through multiple random seeds with appropriate statistical testing is an important direction for future work. Besides, future work should incorporate accession-level validation, extend segmentation to tissue-level resolution (distinguishing xylem, phloem, and sclerenchyma within vascular bundles), and adapt the biologically informed weighting strategy to other crop species.

In summary, this study presents a cost-effective, fully automated pipeline for semantic segmentation of rice stem internal structures. The UNet-ECA-Bio model achieves high segmentation accuracy and demonstrates that model-predicted phenotypes can preserve genetic signals comparable to manual annotation in GWAS. The accompanying open-source software lowers the barrier to large-scale stem phenotyping, providing a practical tool for genetic studies of lodging resistance and yield improvement.

## Data Availability

All datasets and models are downloadable via the following URL: https://doi.org/10.5281/zenodo.21501303.

## Supplementary material

Supplementary Figure 1 Comparative analysis of mIoU/IoU of SVB across varying input sizes.

Supplementary Figure 2 Enhanced UNet Architecture with ECA Attention Mechanism and biologically informed class weights.

Supplementary Figure 3 Prediction of the internal structure of wheat stems.

Supplementary Figure 4 QQ plot of predicted and annotated stem internal structural traits.

Supplementary Figure 5 Clusters of annotated genes based on string database.

Supplementary Table 1 Information of the rice accessions.

Supplementary Table 2 Comparison of different initial learning rates by the UNet-VGG16 model.

Supplementary Table 3 Comparison of different batch size in the UNet-VGG16 model.

Supplementary Table 4 Comparison of different input size in UNet-VGG16 model.

Supplementary Table 5 Comparison of different epochs in UNet-VGG16 model.

Supplementary Table 6 Description of annotated and predicted stem internal structural traits.

Supplementary Table 7 Annotation of significant SNPs of stem internal structural traits.
