# Supplementary material for: UNet-ECA-Bio: a biologically informed deep learning model for high-throughput micro-phenotyping of rice stem vascular bundles
Source: Front Plant Sci. 2026 Jul 28;17:1893490. doi: 10.3389/fpls.2026.1893490 (PMC13457637; doi:10.3389/fpls.2026.1893490)
Supplement: Supplementary file 2 [file DataSheet2.pdf]

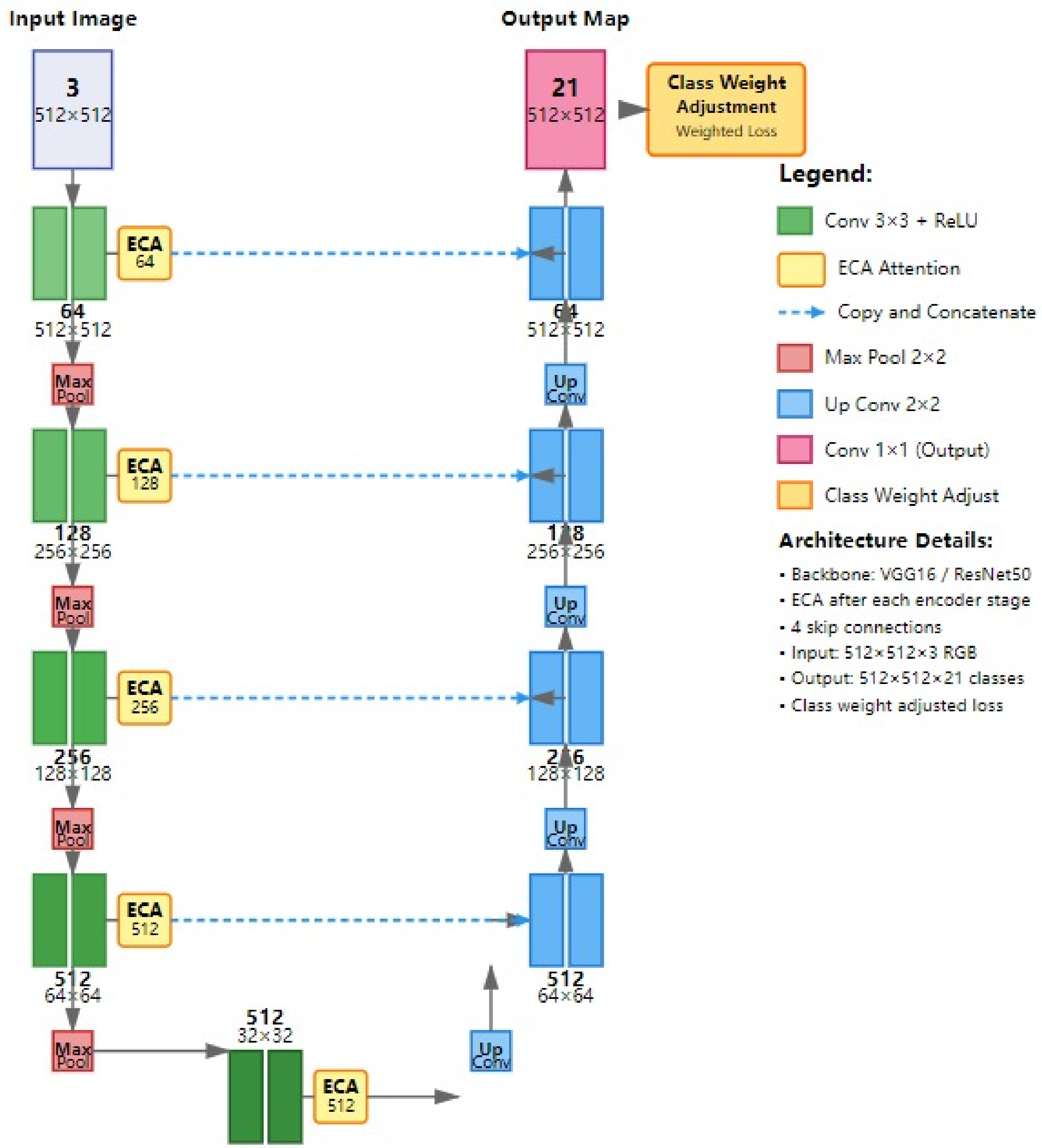

Input Image
3
512×512
64
512×512
ECA
64
Max Pool
128
256×256
ECA
128
256
128×128
ECA
256
512
64×64
ECA
512
512
32×32
ECA
512
Up Conv
Output Map
21
512×512
Class Weight Adjustment
Weighted Loss
Legend:
Conv 3×3 + ReLU
ECA Attention
Copy and Concatenate
Max Pool 2×2
Up Conv 2×2
Conv 1×1 (Output)
Class Weight Adjust
Architecture Details:
• Backbone: VGG16 / ResNet50
• ECA after each encoder stage
• 4 skip connections
• Input: 512×512×3 RGB
• Output: 512×512×21 classes
• Class weight adjusted loss
