# Supplementary material for: UNet-ECA-Bio: a biologically informed deep learning model for high-throughput micro-phenotyping of rice stem vascular bundles
Source: Front Plant Sci. 2026 Jul 28;17:1893490. doi: 10.3389/fpls.2026.1893490 (PMC13457637; doi:10.3389/fpls.2026.1893490)
Supplement: Supplementary file 3 [file DataSheet3.pdf]

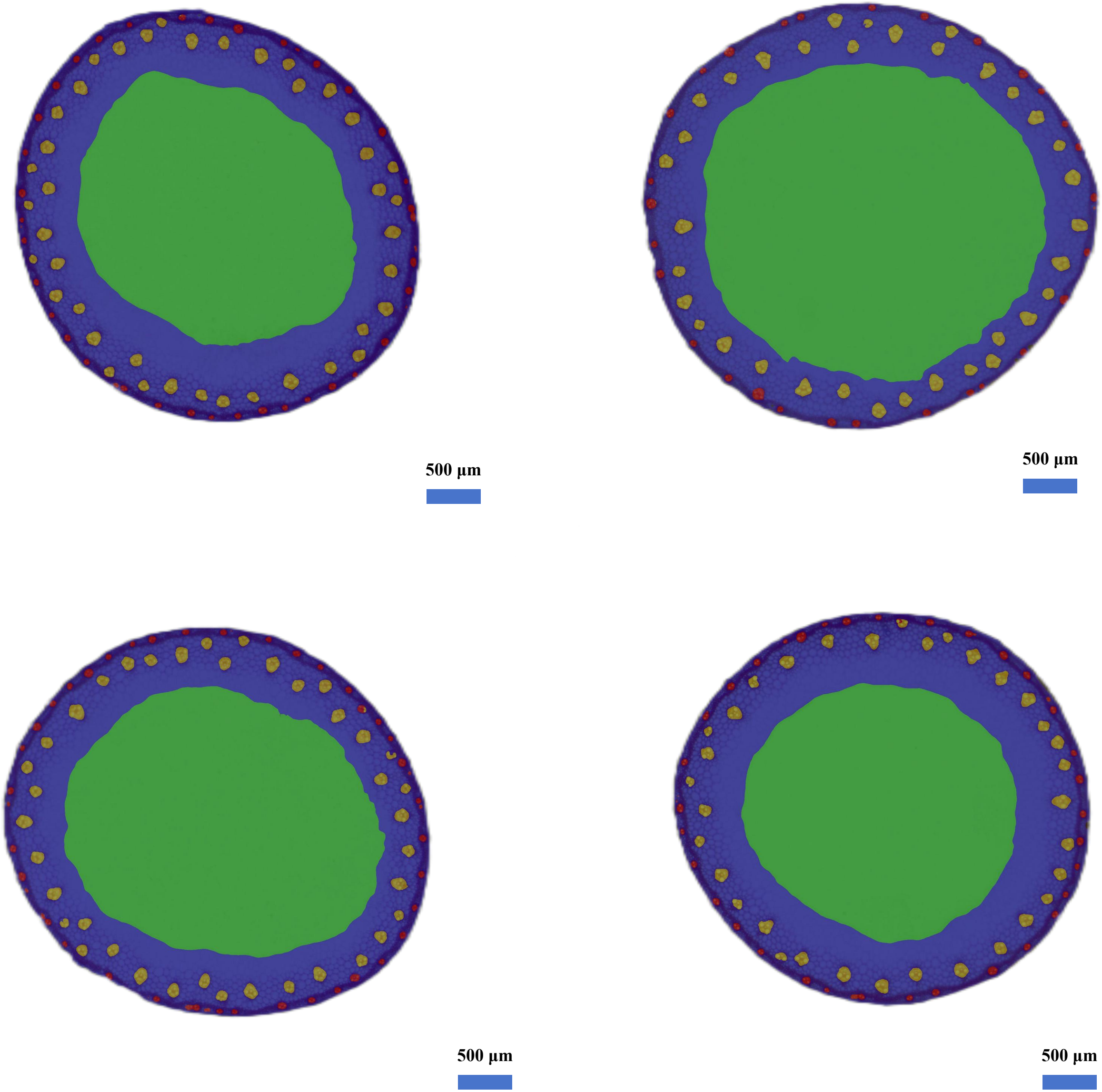

500 µm
500 µm
500 µm
500 µm
