# Supplementary material for: UNet-ECA-Bio: a biologically informed deep learning model for high-throughput micro-phenotyping of rice stem vascular bundles
Source: Front Plant Sci. 2026 Jul 28;17:1893490. doi: 10.3389/fpls.2026.1893490 (PMC13457637; doi:10.3389/fpls.2026.1893490)
Supplement: Supplementary file 4 [file DataSheet4.pdf]

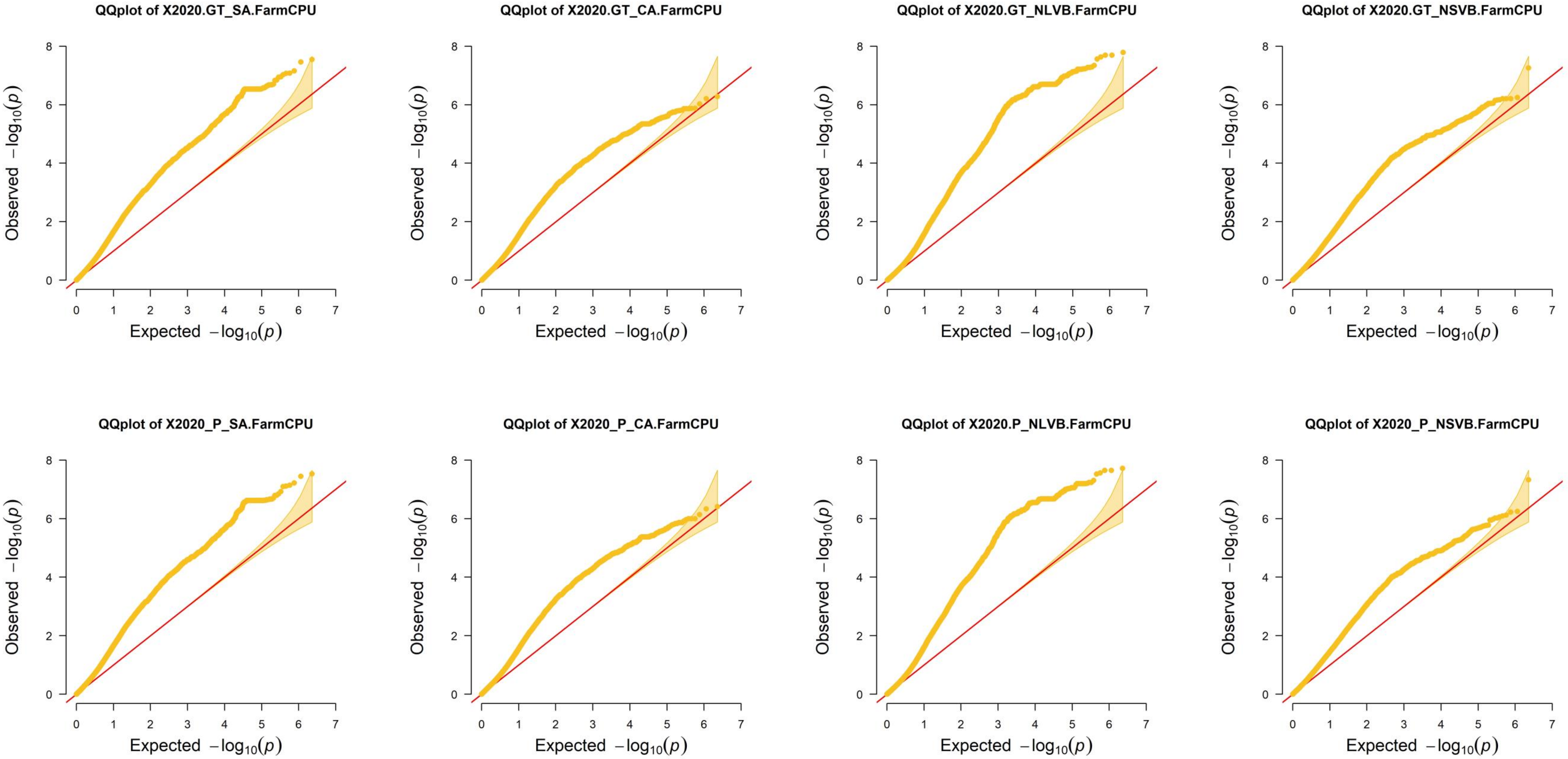
QQplot of X2020.GT_SA.FarmCPU
QQplot of X2020.GT_CA.FarmCPU
QQplot of X2020.GT_NLVB.FarmCPU
QQplot of X2020.GT_NSVB.FarmCPU
QQplot of X2020_P_SA.FarmCPU
QQplot of X2020_P_CA.FarmCPU
QQplot of X2020.P_NLVB.FarmCPU
QQplot of X2020_P_NSVB.FarmCPU
Observed $-\log_{10}(p)$
Expected $-\log_{10}(p)$
0
2
4
6
8
0
1
2
3
4
5
6
7
