# Supplementary material for: UNet-ECA-Bio: a biologically informed deep learning model for high-throughput micro-phenotyping of rice stem vascular bundles
Source: Front Plant Sci. 2026 Jul 28;17:1893490. doi: 10.3389/fpls.2026.1893490 (PMC13457637; doi:10.3389/fpls.2026.1893490)
Supplement: Supplementary file 5 [file DataSheet5.pdf]

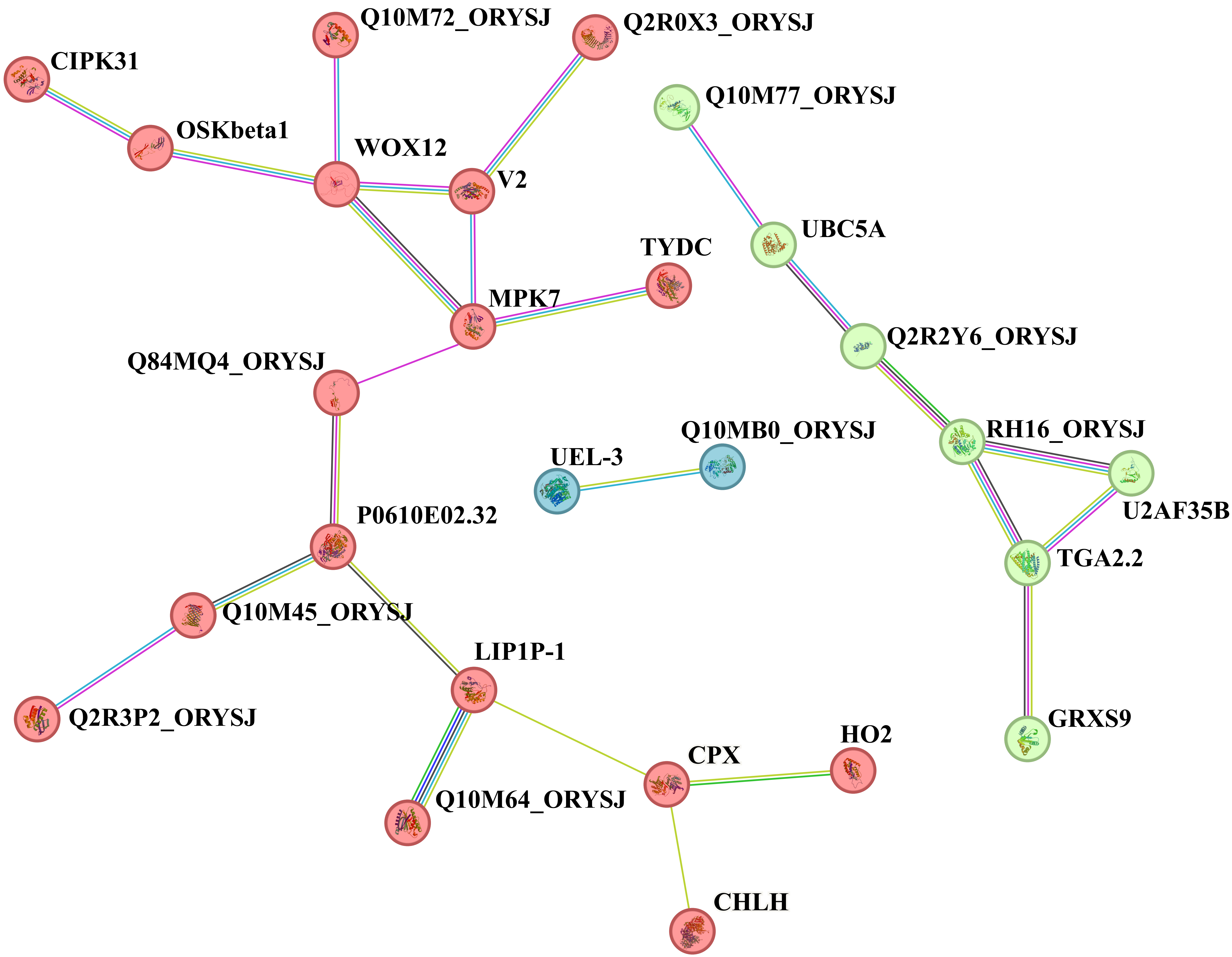

Q10M72_ORYSJ
Q2R0X3_ORYSJ
CIPK31
OSKbeta1
WOX12
V2
Q10M77_ORYSJ
UBC5A
TYDC
MPK7
Q2R2Y6_ORYSJ
Q84MQ4_ORYSJ
RH16_ORYSJ
Q10MB0_ORYSJ
UEL-3
U2AF35B
P0610E02.32
TGA2.2
Q10M45_ORYSJ
LIP1P-1
Q2R3P2_ORYSJ
GRXS9
HO2
CPX
Q10M64_ORYSJ
CHLH
